# Allodynia by Splenocytes From Mice With Acid-Induced Fibromyalgia-Like Generalized Pain and Its Sexual Dimorphic Regulation by Brain Microglia

**DOI:** 10.3389/fnins.2020.600166

**Published:** 2020-12-23

**Authors:** Hiroshi Ueda, Naoki Dozono, Keigo Tanaka, Shuji Kaneko, Hiroyuki Neyama, Hitoshi Uchida

**Affiliations:** ^1^Department of Pharmacology and Therapeutic Innovation, Nagasaki University Institute of Biomedical Sciences, Nagasaki, Japan; ^2^Department of Molecular Pharmacology, Kyoto University Graduate School of Pharmaceutical Sciences, Kyoto, Japan; ^3^RIKEN Center for Biosystems Dynamics Research, Kobe, Japan; ^4^Department of Cellular Neuropathology, Brain Research Institute, Niigata University, Niigata, Japan

**Keywords:** fibromyalgia, sex difference, microglia, T cells, brain-immune, acid-induced allodynia

## Abstract

Fibromyalgia (FM), a disease of unknown etiology characterized by chronic generalized pain, is partly recapitulated in an animal model induced by repeated acid saline injections into the gastrocnemius muscle. Here, we attempted to investigate the sex difference in pain hypersensitivity (mechanical allodynia and hypersensitivity to electrical stimulation) in the repeated acid saline-induced FM-like generalized pain (AcGP) model. The first unilateral acid injection into gastrocnemius muscle at day 0/D0 and second injection at D5 (post day 0, P0) induced transient and long-lasting mechanical allodynia, respectively, on both sides of male and female mice. The pretreatment with gonadectomy did not affect the first injection-induced allodynia in both sexes, but gradually reversed the second injection-induced allodynia in male but not female mice. Moreover, the AcGP in male mice was abolished by intracerebroventricular minocycline treatments during D4–P4 or P5–P11, but not by early treatments during D0–D5 in male but not female mice, suggesting that brain microglia are required for AcGP in late-onset and sex-dependent manners. We also found that the intravenous treatments of splenocytes derived from male but not female mice treated with AcGP caused allodynia in naive mice. In addition, the purified CD4^+^ T cells derived from splenocytes of acid-treated male mice retained the ability to cause allodynia in naive mice. These findings suggest that FM-like AcGP has multiple sexual dimorphic mechanisms.

## Introduction

During the past three decades, our understanding of mechanisms for neuropathic pain (NeuP) has enormously advanced, primarily through application of molecular, genetic, anatomical, and physiological techniques to the study of animal models of NeuP. For instance, following peripheral nerve injury, peripheral and central neurons are known to display multiple alterations in their functions and structures in a spatiotemporal manner and cause chronic NeuP (Scholz and Woolf, 2007; Costigan et al., 2009b; Ji et al., 2016; Kuner and Flor, 2016; Ueda, 2017; Chen et al., 2018). Recently, many researchers focus on the neuroinflammation caused by infiltration of immune cells, activation of glial cells, and the factors from these cells due to its pathophysiological roles (Costigan et al., 2009a; Salter and Stevens, 2017; Chen et al., 2018; Ji et al., 2019; Yu et al., 2020). More importantly, recent elegant studies have clarified the presence of sexual dimorphism in the mechanisms underlying the neuroinflammation in animal models of NeuP (Mapplebeck et al., 2016, 2017; Boerner et al., 2018; Mogil, 2020). They showed that microglial cells and macrophages are required for NeuP in male but not female mice, whereas T lymphocytes and related molecules are likely to be alternatively utilized in female mice (Sorge et al., 2011, 2015; Mapplebeck et al., 2016; Luo et al., 2019). Such sex difference in the involvement of immune system is one of the most important issues to be addressed in future studies for the drug discovery.

Compared to NeuP, the mechanisms underlying female-predominant fibromyalgia (FM), a disease of unknown etiology that leads to chronic widespread pain with high prevalence in the general population, are much less understood (Clauw, 2014; Wolfe et al., 2014; Häuser et al., 2015). The prevalence ratio of NeuP in the general population is reported to be 3–17% (van Hecke et al., 2014), while that of FM is reported to be 2.0% for both sexes, 3.4% for women, and 0.5% for men (Wolfe et al., 1995). Along with the revision of FM diagnosis criteria, the nature of generalized pain with regional definition and symptom severity has been currently used for the diagnosis rather than the previously utilized muscle pain or widespread pain based on tender point definition (Wolfe et al., 1990, 2010, 2011, 2016). Since FM has multiple risk factors, including stress, genetic polymorphisms, and familial predisposition (Häuser et al., 2017), the investigation of multiple animal models developed by a wide variety of methods is therefore a proper strategy for the development of diagnosis and treatments. To date, based on the symptomatology and possible pathogenesis, animal models of FM have been developed by using repeated muscle insult or exposure to physical and psychological stresses. Khasar et al. (1998) demonstrated that vagotomized rats show widespread pain (Khasar et al., 1998) and Sluka et al. (2001) developed acid saline-induced generalized pain (AcGP; Sluka et al., 2001). We have then developed intermittent cold stress (ICS) or intermittent psychological stress (IPS)-induced generalized pain disease models (Nishiyori and Ueda, 2008; Ueda and Neyama, 2017). Other models were developed by use of intermittent sound stress and reserpine administration-induced models (Khasar et al., 2009; Nagakura et al., 2009). Most of these models have evidenced that the chronic widespread pain is sensitive to antidepressants and gabapentinoids, but not to nonsteroidal anti-inflammatory drugs, as seen in FM patients (Clauw, 2014). Limited information is available about the sex difference in ICS and IPS models, where the allodynia was reversed in orchiectomy (ORX)-treated male mice, but not in ovariectomy (OVX)-treated female ones (Nishiyori and Ueda, 2008; Ueda and Neyama, 2017). Here, we report the first evidence of sex difference in terms of the involvement of microglia and peripheral immune cells in the AcGP model.

## Materials and Methods

### Animals

A total of 357 male (7–10 weeks, 16–25 *g*) and female (7–10 weeks, 16–20 *g*) C57BL/6J JmsSlc mice from Nihon SLC (Shizuoka, Japan) were used. They were kept in a room maintained at 24 ± 1°C and 55 ± 10% relative humidity with a 12-h light/dark cycle, and had free access to a standard laboratory diet and tap water.

### AcGP Model

According to the previous paper (Sluka et al., 2001), mice were treated with acid saline. Briefly, under the isoflurane (4%) anesthesia, mice were unilaterally injected twice 5 days apart (Day 0/D0 and Day 5/D5) with 20 μl of pH 4.0 saline into the gastrocnemius muscle of the right or left hindlimb, by using a 27-gage needle. Post day 1 (P1) represents day 1 after the second acid injection.

### Gonadectomy

Gonadectomy (ORX and OVX) has been performed according to the widely utilized protocol (Sophocleous and Idris, 2019). After the surgery, mice were kept in a soft bed cage with some food inside so that the animals could feed themselves without difficulty in standing. 3 weeks after the ORX or OVX, the first acid saline injection was performed.

### Drug Treatment

Minocycline hydrochloride (Sigma-Aldrich, St. Louis, MO, United States) was dissolved in the artificial cerebrospinal fluid (aCSF; 125 mM NaCl, 3.8 mM KCl, 1.2 mM KH_2_PO_4_, 26 mM NaHCO_3_, and 10 mM glucose, pH 7.4) and injected intracerebroventricularly (i.c.v.) into the right lateral ventricle of conscious mice at a dose of 10 nmol/5 μl, according to the established method (Haley and McCormick, 1957). Minocycline was treated during the 1st (D0–D5), 2nd (D4–P4), or 3rd (P5–P11) stage. Tacrolimus obtained from Fujisawa Pharmaceutical (Osaka, Japan) was dissolved in the solution containing 10% dimethyl sulfoxide and 90% corn oil, and administrated intraperitoneally at a dose of 10 mg/kg (0.1 ml/10 *g* body weight) 30 min prior to the second acid saline injection. Solvents were used for vehicle control.

### Nociception Tests

In the mechanical paw pressure test, mice were placed in a plexiglass chamber on a 6 × 6 mm wire mesh grid floor and allowed to acclimatize for a period of 1 h, as reported (Neyama et al., 2020). A mechanical pain stimulus was then delivered to the middle of the plantar surface of the right hind paw using an electronic digital von Frey anesthesiometer and rigid tip (Model 2390, 90 *g* probe; IITC Inc., Woodland Hills, CA, United States). The pain threshold was evaluated by the pressure needed to induce a paw flexor response.

An electrical stimulation-induced paw withdrawal test (EPW) was performed as described previously (Matsumoto et al., 2008; Ueda, 2017; Neyama et al., 2020). In this test, electrodes of a Neurometer Current Perception Threshold/C (CPT/C; Neurotron Inc., Baltimore, MD, United States) were fastened to the planter and the insteps of the hind paw. Transcutaneous nerve stimuli consist of each of the three sine-wave pulses (5, 250, and 2,000 Hz), and the minimum intensity at which each mouse withdrew its paw (cutoff time: 3 s) was evaluated as the nociceptive current threshold. In a previous paper (Koga et al., 2005), the durations of single sine-wave stimuli at frequencies of 250 and 2,000 Hz were 4 and 0.5 ms, respectively, while no information of duration of 5 Hz was available.

### Preparation of Single Cell Suspension From the Spleen

The spleen isolated from acid- or vehicle-treated mouse was put into a sterile six-well dish in 3 ml of ice-cold RPMI 1640 medium (Gibco, Grand Island, NY, United States), containing 2% fetal bovine serum (FBS) and minced by use of the plunger of a 1-ml injection syringe. Dissociated tissue was transferred to the mesh strainer on a 50-ml tube, gently passed through the mesh strainer of 70 μm pore size (Corning, Glendale, AZ, United States), and washed by 5 ml of ice-cold RPMI containing 2% FBS. Dissociated splenocytes were centrifuged 500 × *g* for 5 min at 4°C and the supernatant was discarded. The pellet was incubated with 3 ml of red blood cell lysis buffer (Abcam, San Diego, CA, United States) for 3 min at room temperature. The hemolysis was stopped by adding 5 ml of ice-cold PBS containing 2% FBS, and the cells were centrifuged at 500 × *g* for 5 min at 4°C and the supernatant was discarded. The pellet was washed by 5 ml of ice-cold PBS containing 2% FBS, resuspended in 3 ml of the same buffer, and passed through the mesh strainer. After counting the cell number, aliquots of splenocytes were administered into the right retro-orbital sinus of the naïve mouse under the isoflurane (4%) anesthesia (Yardeni et al., 2011). In the present study, we used 200 μl of injection volume, aiming the efficient intravenous delivery of cell suspensions, but we experienced no significant difference from the case using 150 μl, which is recommended by the previous report (Yardeni et al., 2011).

### Preparation of CD4^+^ Cells From Splenocytes

To isolate CD4^+^ cells, the suspension of splenocytes (1 × 10^7^ cells) was labeled with 10 μl of anti-CD4 (L3T4) Microbeads (Miltenyi Biotec, Bergisch Gladbach, Germany) at 4°C for 10 min. Thereafter, the labeled cells were separated into cell fractions that were positive or negative for CD4 by using MACS column, according to the manufacturer’s protocol (Miltenyi Biotec). We obtained 3 × 10^5^ CD4^+^ cells from 1 × 10^7^ of splenocytes. The isolated cells were further diluted 1:10 in PEB buffer [PBS, pH 7.2, 0.5% bovine serum albumin, and 2 mM EDTA; prepared by diluting MACS BSA Stock Solution 1:20 with autoMACS^®^ Rinsing Solution (Miltenyi Biotec)] and used for intravenous injection into naive mice.

### Statistical Analysis

All data are shown as mean ± S.E.M. Statistical value was calculated by GraphPad Prism 7 (GraphPad Software Inc., La Jolla, CA, United States), with *P* value set at 0.05 and 0.01. The normality was assessed by using Shapiro–Wilk normality test. Statistical differences between the group were analyzed using two-way repeated measures ANOVA followed by Bonferroni’s, Tukey’s, or Dunnett’s multiple comparisons test or one way ANOVA followed by Tukey’s multiple comparisons test. Individual statistics was presented in figure legends.

## Results

### Long-Lasting Bilateral Mechanical Allodynia Induced by Repeated Acid Injections Into the Gastrocnemius Muscle

**FIGURE 1:**
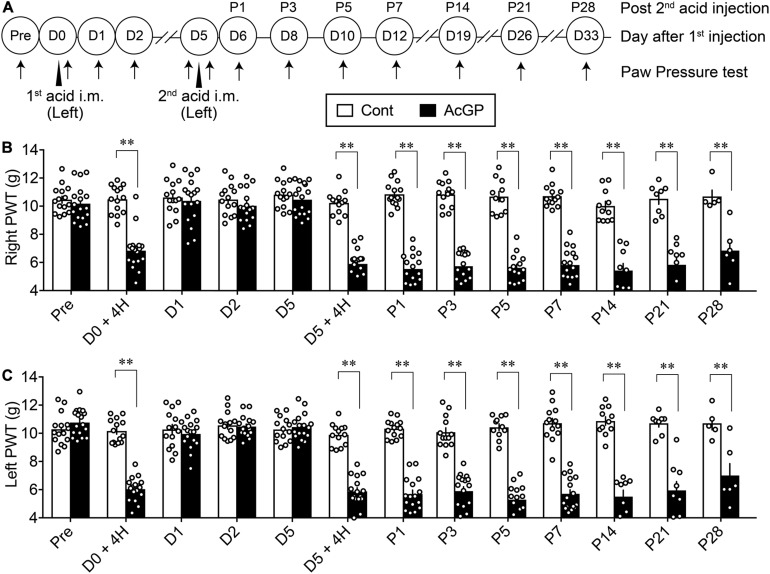
Bilateral mechanical allodynia by intramuscular injections of acid saline. **(A)** Twenty microliters of acid saline (pH 4.0) or control saline (pH 7.2) was injected into the “Left” gastrocnemius muscle twice 5 days apart, as indicated by arrowheads. Mechanical paw pressure tests on both sides were performed at the indicated time points (arrows). The day for the first acid saline injection is indicated as day 0/D0, and the next day after the second acid saline injection is indicated as P1. **(B,C)** Mechanical allodynia on the contralateral (right, **B**) and ipsilateral side (left, **C**) following the acid saline injections into the left gastrocnemius muscle at D0 and D5. The transient and long-lasting allodynia following first and second acid saline, respectively, were evaluated by the paw pressure test on both sides. ^∗∗^*p* < 0.01, compared with vehicle-treated control (Cont) mice at each time point, in two-way repeated measures ANOVA followed by Bonferroni’s multiple comparisons test (Cont *n* = 5–15, AcGP *n* = 6–17). The dots in the column represent number of animals. AcGP, acid saline-induced FM-like generalized pain; PWT, paw withdrawal threshold.

Male mice were injected twice 5 days apart (Day 0/D0 and D5) with 20 μl of pH 4.0 saline into the gastrocnemius muscle of the left hindlimb (Figure 1A). The first acid injection significantly decreased the paw withdrawal threshold on both right and left sides at 4 h, while the threshold on both sides was returned to the basal level at 24 h post-injection (D1) and then sustained throughout D5, as shown in Figures 1B,C. On the other hand, the second acid injection at D5 also decreased the allodynia at 4 h, and the allodynia persisted for longer than 28 days after the second acid injection (Figures 1B,C). In contrast, repeated injections of control saline (pH 7.2) had no effects on mechanical pain thresholds on both sides (Figures 1B,C).

### Male-Specific and Time-Dependent Recovery From the Mechanical Allodynia Following Acid Injection Into the Right Muscle of Gonadectomized Mice

**FIGURE 2:**
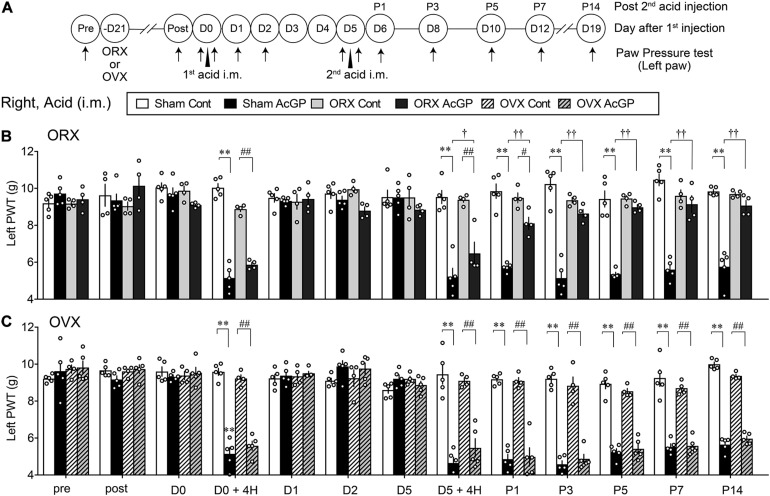
Male-specific blockade of contralateral AcGP by gonadectomy. **(A)** The surgical operation of orchiectomy or ovariectomy was performed 3 weeks before the first acid saline injection. Time points of acid saline injection into “Right” muscle and paw pressure test at the left paw were indicated by arrowheads and arrows, respectively. **(B,C)** Sexual dimorphic effects of ORX **(B)** and OVX **(C)** on the long-lasting mechanical allodynia after the second acid saline injection. Results represent the nociceptive paw withdrawal threshold (PWT in g) on the left side in the paw pressure test after the right acid saline injection (i.m.) in sham-operated or gonadal removed mice. Data at “post” were obtained at the day of 3 weeks after the gonadectomy. ^∗∗^*p* < 0.01, compared with each time point at Sham Cont, ^#^*p* < 0.05; ^##^*p* < 0.01, compared with each time point at ORX Cont **(B)** or OVX Cont **(C)**, ^†^*p* < 0.05; ^†⁣†^*p* < 0.01, compared with each time point at Sham AcGP, in two-way repeated measures ANOVA followed by Tukey’s multiple comparisons test (Sham Cont, *n* = 4–5; Sham AcGP, *n* = 5; ORX Cont, *n* = 4, ORX Acid, *n* = 4; OVX Cont, *n* = 4; and OVX AcGP, *n* = 5). The dots in the column represent number of animals. AcGP, acid saline-induced FM-like generalized pain; ORX, orchiectomy; OVX, ovariectomy.

As in the case with the acid injection into the left muscle, the repeated acid injections into the right muscle produced transient allodynia and long-lasting allodynia after the first and second acid injection, respectively, and there was no significant sex difference (Figures 2A–C). To investigate the effect of gonadectomy on acid saline-induced mechanical allodynia, the surgery of ORX or OVX was made 3 weeks before the first injection. These surgeries had no effects on the mechanical pain thresholds and the transient allodynia after the first acid injection (Figures 2B,C). We found that ORX significantly reduced the mechanical allodynia after the second acid injection (Figure 2B). The attenuation was started at as early as 4 h and significant loss of allodynia was at P1 and later through P5. Unlike ORX, OVX did not attenuate the second acid-induced allodynia (Figures 2B,C). Quite similar results were also observed when mechanical nociception tests were performed to the ipsilateral (right) paw (Supplementary Figure 1).

### Lack of Preventive Action by the Early-Stage Minocycline Treatments on AcGP

**FIGURE 3:**
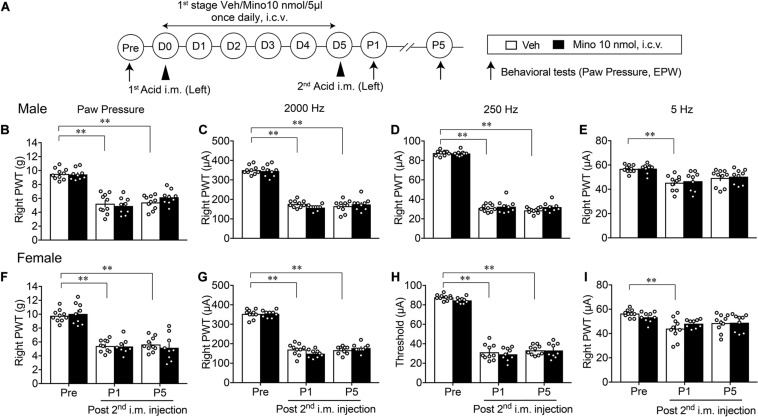
Lack of effects by early treatments with brain minocycline on the AcGP in male and female mice. **(A)** Minocycline dissolved in 10 nmol/5 μl of aCSF or aCSF (as a vehicle, Veh) was administered (i.c.v.) once daily from D0 to D5, as the 1st-stage treatments. Behavioral tests represent the paw pressure test and EPW test using 2,000, 250, and 5 Hz electrical stimulation **(B–I)** Sexual dimorphic effects by 1st stage of minocycline treatments (D0–D5) on the abnormal pain behaviors induced by acid saline into the left muscle. Results represent the nociceptive threshold (g) on the right side at indicated time points (arrows) in the paw pressure test **(B,F)** and the threshold (μA) in the EPW test using 2000 Hz **(C,G)**, 250 Hz **(D,H)**, and 5 Hz **(E,I)**, respectively, in male **(B–E)** and female **(F–I)** mice. ^∗∗^*p* < 0.01, compared with Pre, in two-way repeated measures ANOVA followed by Dunnett’s multiple comparisons test (Veh, *n* = 10; Male Mino, *n* = 9; Female Mino, *n* = 8–9). The dots in the column represent number of animals. AcGP, acid saline-induced FM-like generalized pain; Mino, minocycline; and PWT, paw withdrawal threshold.

We investigated the involvement of brain microglia on AcGP, by using minocycline, a representative inhibitor of microglia actions. As shown in Figure 3A, minocycline was treated daily for 6 days (D0–D5, 1st-stage treatments) starting with the first administration through an i.c.v. route at 30 min before the acid saline injection into the left muscle. In this paradigm of study, nociceptive threshold was evaluated in the EPW test using 2,000, 250, and 5 Hz electrical stimulation, which activates Aβ-, Aδ-, and C-fibers, respectively (Koga et al., 2005), on the right hind paw as well as mechanical paw pressure test. In male mice, the AcGP in terms of mechanical allodynia at P1 and P5 was not affected by the 1st-stage treatments with minocycline (10 nmol), as shown in Figures 3B–E. On the other hand, the thresholds of 2,000 and 250 Hz but not 5 Hz electrical currents to cause nociceptive paw withdrawal responses were significantly decreased by repeated acid saline treatments, while the 1st-stage minocycline treatments had no effects on them. Quite similar results were also observed in female mice (Figures 3F–I).

### Male-Specific Blockade of the Development of AcGP by the Late-Stage Minocycline Treatments

**FIGURE 4:**
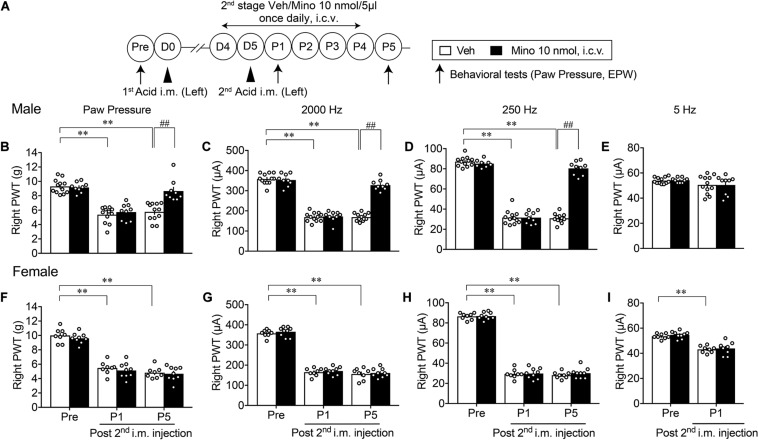
Effective blockade of AcGP by 2nd stage of minocycline treatments. Details are described in the legend of Figure 3, except for the minocycline treatments (D4 to P4; **A**). For **(B–D)** and **(F–H)**, ^∗∗^*p* < 0.01, compared with Pre, two-way ANOVA followed by Dunnett’s multiple comparisons test. ^##^*p* < 0.01, compared with each time point at Veh, two-way ANOVA followed by Bonferroni’s multiple comparisons test. For **(I)**, ^∗∗^*p* < 0.01, compared with basal threshold prior to vehicle injection (Pre Veh), in two-way repeated measures ANOVA followed by Bonferroni’s multiple comparisons test (Male Veh, *n* = 12; Mino, *n* = 9; Female, *n* = 8; and Mino, *n* = 9). For **(E)**, no significant difference by AcGP or minocycline-treatment was observed. The dots in the column represent number of animals. AcGP, acid saline-induced FM-like generalized pain; Mino, minocycline; and PWT, paw withdrawal threshold.

A similar paradigm of experiments was performed except for the 2nd-stage minocycline treatments (D4–P4), as shown in Figure 4A. Although there were no effects on the mechanical allodynia and nociceptive sensitization by 2,000 and 250 Hz but not 5 Hz electrical stimulation at P1 in male mice, significant recovery of these abnormal pain or pain-related behaviors was observed at P5 (Figures 4B–E). However, no significant recovery of abnormal pain-related behaviors at P1 and P5 was observed in female mice (Figures 4F–I).

**FIGURE 5:**
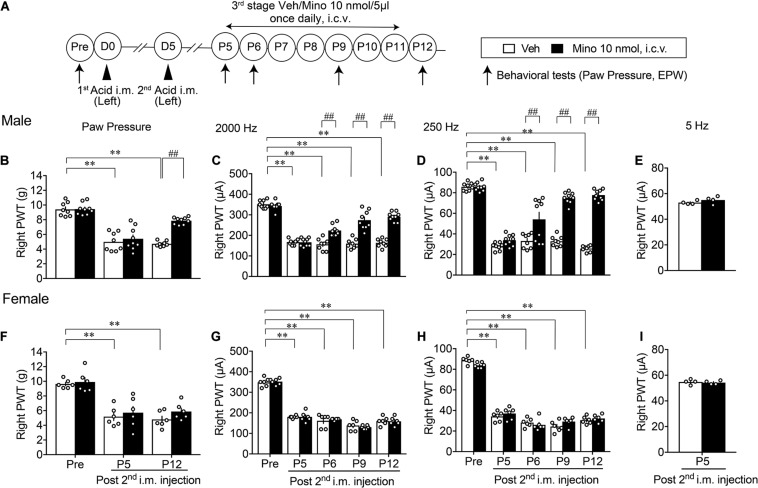
Effective blockade of AcGP by 3rd stage of minocycline treatments. Details are described in the legend of Figure 3, except for the minocycline treatments (P5 to P11; **A**). For **(B–D)** and **(F–H)**, ^∗∗^*p* < 0.01, compared with basal threshold prior to vehicle injection (Pre Veh), in two-way repeated measures ANOVA followed by Dunnett’s multiple comparisons test. ^##^*p* < 0.01, compared with each time point at Veh, in two-way repeated measurement ANOVA followed by Bonferroni’s multiple comparisons test (Male Veh, *n* = 8; Mino, *n* = 9; and Female, *n* = 6). For **(E,I)**, *P* = 0.2666 **(E)**, and *P* = 0.7084 **(I)**, compared with Mino 10 nmol, i.c.v., in unpaired *t* test (*n* = 4). The dots in the column represent number of animals. AcGP, acid saline-induced FM-like generalized pain; Mino, minocycline; and PWT, paw withdrawal threshold.

When minocycline treatments were given at the later 3rd stage (P5–P11, Figure 5A), the abnormal mechanical allodynia at P12 was significantly reversed in male mice (Figure 5B). Similarly, the hypersensitivity to 2,000 and 250 Hz but not 5 Hz stimulation was time-dependently reversed by repeated minocycline treatments (Figures 5C–E). However, no significant recovery of abnormal pain-related behaviors, such as the mechanical allodynia at P12 and the hypersensitivity to 2,000 and 250 Hz stimulation, was observed in female mice (Figures 5F–H). The withdrawal threshold for 5 Hz stimulation at P5 remained unchanged by minocycline treatments (Figure 5I).

### Reproduction of Allodynia by Splenocytes Derived From Mice With AcGP and Its Blockade by Brain Minocycline Pretreatments in Male Mice

When tacrolimus (10 mg/kg, i.p.) was given 30 min before the second acid injection into the left muscle in male mice (Supplementary Figure 2A), the mechanical allodynia on the right paw at P5 in the AcGP model was significantly reversed (Supplementary Figure 2B). As this preliminary finding suggested that the peripheral immune system may contribute to the AcGP, we attempted to see effects of splenocytes from donor mice with AcGP. When splenocytes were prepared from male mice with AcGP at P5 and intravenously (i.v.) administered to naive male mice in various amounts of splenocytes, the abnormal hypersensitive behaviors to 2,000 and 250 Hz electrical stimuli at day 1 after injection were observed in a cell number-dependent fashion. The approximate number of cells to show maximal effects was 1 × 10^6^ cells (Supplementary Figure 3).

**FIGURE 6:**
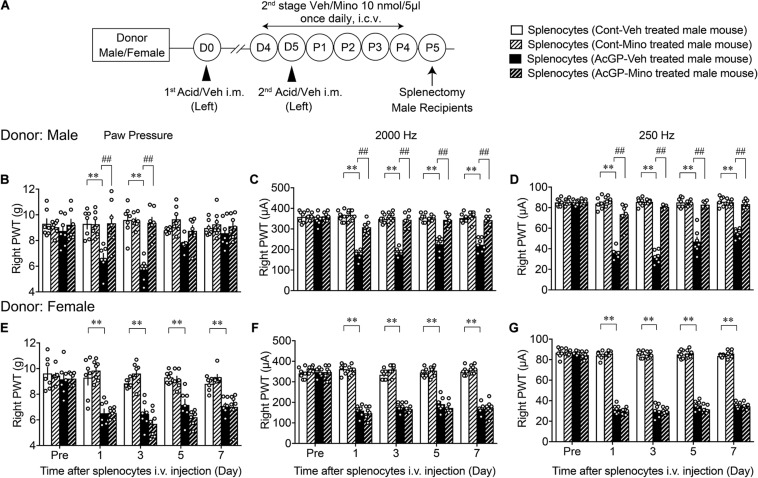
Allodynia by splenocytes derived from mice with AcGP and its blockade by brain minocycline pretreatments in male mice. **(A)** Experimental design. **(B–G)** Allodynia by i.v. injection of splenocytes from male and female mice with AcGP with or without minocycline treatments. Splenocytes prepared from male **(B–D)** or female **(E–G)** mice with control or acid saline injection (left) were i.v. injected into naive male mice. Paw pressure and EPW tests were performed on the right side after the splenocyte injection. The 2nd stage of vehicle or minocycline treatments was given to donor male or female mice. ^∗∗^*p* < 0.01, compared with Pre Veh, in two-way repeated measures ANOVA followed by Dunnett’s multiple comparisons test. ^##^*p* < 0.01, compared with each time point at splenocytes (AcGP-Veh-treated mouse), in two-way repeated measurement ANOVA followed by Tukey’s multiple comparisons test (*n* = 7). The dots in the column represent number of animals. AcGP, acid saline-induced FM-like generalized pain; PWT, paw withdrawal threshold.

Based on these preliminary studies, we next examined whether splenocytes from sensitized mice could be regulated by the pain memory system, which is associated with brain microglia activity (Figure 6A). In this study, splenocytes were prepared from male donor mice, which had been treated with control or AcGP paradigm with 2nd-stage vehicle or minocycline treatments. Splenocytes (1 × 10^6^ cells) derived from male mice treated with AcGP paradigm caused mechanical allodynia and pain-related hypersensitivity to 2,000 and 250 Hz electrical stimulation. The peak effects were observed at day 1 or 3 after i.v. administration, as shown in Figures 6B–D. After the peak effect, mechanical allodynia was gradually reversed to the level with splenocytes from control mice at day 7, while the recovery from the hypersensitivity to 2,000 and 250 Hz electrical stimulation was little slower than the case with mechanical allodynia, suggesting the sensory fiber-specific difference of sensitivity to activated splenocytes or related chemical mediators. Splenocytes from male mice treated with AcGP paradigm and 2nd-stage minocycline administrations showed no significant mechanical allodynia or hypersensitivity to 2,000 and 250 Hz electrical stimulation (Figures 6B–D).

On the other hand, splenocytes derived from female mice treated with AcGP paradigm also caused mechanical allodynia, but no significant recovery from the allodynia was observed throughout day 7 (Figure 6E). The blockade of mechanical allodynia by minocycline pretreatments was not observed, but rather stronger allodynia action was observed at day 3. In the EPW test using 2,000 and 250 Hz, splenocytes from female mice with and without minocycline pretreatments showed the constant hypersensitivity throughout day 7 (Figures 6F,G).

### Crucial Roles of Splenic CD4^+^-Cells in the Reproduction of Allodynia

**FIGURE 7:**
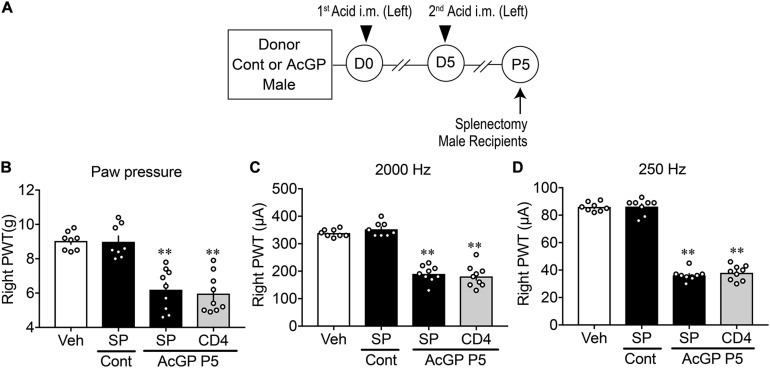
Reproduction of allodynia by splenic CD4^+^-cells. **(A)** Experimental design. Splenocytes were isolated at P5 and then dissociated and separated by MACS beads with anti-CD4 or anti-F4/80 antibody. Splenocytes and separated cells were used for the study of gene expression and injection to naive male mice for paw pressure and EPW tests. **(B–D)** Allodynia by splenic CD4^+^ T cells. Results represent the mechanical allodynia **(B)**, and hypersensitivity to electrical stimulation of 2,000 Hz **(C)** and 250 Hz **(D)** on the right side of naive male mice at day 1 after cell injection. **(B–D)** ^∗∗^*p* < 0.01, compared with splenocytes (SP) Cont, in one-way ANOVA followed by Tukey’s multiple comparisons test (*n* = 8–9). The dots in the column represent number of animals. Veh, the results in naive mice given PBS, which was used for the suspension of splenocytes or purified cells; SP and CD4: splenocytes and CD4^+^ cells, respectively; Cont and AcGP: the treatments with control (pH 7.2) and acid saline (pH 4.0) in donor male mice. AcGP, acid saline-induced FM-like generalized pain; PWT, paw withdrawal threshold.

To examine whether T cells derived from mice with AcGP could play key roles in the reproduction of abnormal pain behaviors, CD^+^ cells were separated from acid injection-treated male mice-derived splenocytes by use of MACS beads (Figure 7A). The ratio of CD4 transcription in CD4^+^ cells was 5.35 times increased, compared to the ratio in original splenocytes, whereas the ratios of CD8, F4/80, and Ly-6G were decreased to 0.15, 1.04, and 0.04 times, respectively (Supplementary Figure 4).

In the paw pressure and EPW tests, there was no significant difference between the mechanical nociceptive threshold in male mice treated with vehicle (i.v., PBS supplemented with 2% FBS), which was used for the cell suspension, and the suspension of splenocytes from male control mice without acid injections, as shown in Figures 7B–D. The i.v. injection of splenocytes (1 × 10^6^ cells) derived from AcGP mice into naive mice significantly decreased the nociceptive threshold in paw pressure and EPW tests using 2,000 and 250 Hz stimuli (Figures 7B–D). The injection of 10% of total CD4^+^ cells (1 × 10^6^ splenocytes equivalent), which had been separated from original 1 × 10^7^ splenocytes by MACS beads, caused an equivalent allodynia in all three nociception tests in naïve mice (Figures 7B–D).

## Discussion

Firstly, we successfully confirmed that AcGP model displayed a transient bilateral mechanical allodynia 4 h after the first acid injection and the persistent bilateral mechanical allodynia at least for 28 days after the second injection (Figures 3A,B), as reported previously (Sluka et al., 2001). As shown in Figure 4 and Supplementary Figure 1, there was a sexual dimorphic change of mechanical allodynia after the gonadectomy. The allodynia after the second injection on both ipsi- and contralateral sides was gradually reversed by ORX, but not OVX. Similar observations have been found in ICS- or IPS-induced generalized pain disease models, which are closely related to brain functional disturbance through autonomic nervous and emotional stress, respectively (Nishiyori and Ueda, 2008; Ueda and Neyama, 2017). However, the mechanisms underlying male-specific recovery after the second acid treatment remain elusive, but the androgen may be involved in the maintenance of bilateral allodynia, presumably through brain or systemic mechanisms. For instance, Sorge, Mogil, and their colleagues have demonstrated pioneering findings that testosterone plays roles in the emergence of allodynia in a male-specific manner (Sorge et al., 2011). In contrast, a recent paper has shown that the administration of testosterone attenuated the development of widespread muscle pain (Lesnak et al., 2020). Therefore, further studies are required to elucidate the role of sex hormones in generalized pain.

Another type of sexual dimorphism has recently been reported in relation to the spinal microglial function in NeuP models, where the nerve injury-induced NeuP in male rodents is markedly inhibited by microglia ablation or inhibition by specific chemical tools, such as Mac-1-saporin or minocycline treatments, respectively, while the NeuP in female animals is not (Sorge et al., 2015). It is important to note that brain microglia activation is observed in the positron emission tomography study of FM patients (Albrecht et al., 2019). Therefore, we here attempted to see the involvement of brain microglia on AcGP in mice, by using three different types of minocycline treatments, 1st stage of minocycline (D0–D5), 2nd stage (D4–P4), and 3rd stage (P5–P11), which are intended to see microglial roles in the priming, development, and maintenance mechanisms underlying chronic pain, respectively. Moreover, in addition to the conventional mechanical paw withdrawal test, we adopted EPW test to more precisely investigate the effects of minocycline on sensory fiber-specific abnormal pain behaviors after acid injection. In the EPW test, we applied three different sine-wave electrical pulses at 2,000, 250, and 5 Hz, which have been previously characterized to stimulate Aβ-, Aδ-, and C-fibers, respectively, in the studies of electrical physiology (Koga et al., 2005; Matsumoto et al., 2008; Ueda, 2008, 2017). The specificity was also supported by the studies of functional histochemistry and pharmacology, in which spinal phosphorylation of extracellular signal-regulated kinase 1/2 was observed by 5 and 250 Hz but not 2,000 Hz stimulation (Koga et al., 2005; Matsumoto et al., 2008; Ueda, 2008, 2017) and nociceptive responses were differentially blocked by neonatal capsaicin treatment and spinal NMDA or non-NMDA receptor antagonist (Matsumoto et al., 2006). In peripheral NeuP models, there were apparent hypersensitivity to 2000 and 250 Hz stimuli as well as hyposensitivity to 5 Hz stimuli, possibly due to retraction of C-fiber (substance P) neurons at the dorsal horn of spinal cord (Inoue et al., 2006).

As shown in Figures 5–7, the 2nd and 3rd stage of minocycline treatments abolished the acid injection-induced mechanical allodynia and hypersensitivity to Aβ and Aδ stimulation in male, but not female mice, though the 1st stage of minocycline did not affect the AcGP in both sexes. These results are consistent with the case in the peripheral nerve injury-induced NeuP (Sorge et al., 2015). It should be noted that microglia in male mice play roles in the development and maintenance of long-lasting AcGP, but not in the priming stage. In other words, microglial roles in AcGP mechanisms in male mice may be secondary to other unknown mechanisms, which are not driven in female mice. In the present study, in order to examine the sex- and time stage-specific actions of minocycline, we used repeated i.c.v. treatments with minocycline for 6–7 days at a slightly lower dose (10 nmol) than the report in the NeuP model (∼20 nmol, 6 days), which shows the blockade of enhanced Iba-1-immunoreactivity at the anterior cingulate cortex by minocycline (Miyamoto et al., 2017). In our immunohistochemical survey with anti-Iba1 antibody, we failed to detect marked morphological changes of microglia in pain- and stress-related brain regions, including anterior nucleus of paraventricular thalamus, paraventricular hypothalamus, and central nucleus of amygdala. Further studies to evaluate the functional changes in terms of the gene expression in microglia, which can be separated from brain tissues using MACS beads, would be the next subject. Furthermore, we should also perform the study to examine whether spinal cord microglia are involved in the AcGP and the sexual dimorphism exist.

Recent studies have provided evidence for sexual dimorphism in the contribution of immune cells to NeuP (Mapplebeck et al., 2016). For instance, accumulating evidence suggest that female mice may use infiltrating T cells, instead of microglia, in NeuP (Sorge et al., 2015). Also, Toll-like receptor 9 in macrophage is involved in chemotherapy-induced peripheral neuropathy in male but not female mice (Luo et al., 2019). Thus, we attempted to examine whether AcGP is affected by the treatment with tacrolimus, a representative immunosuppressant. As shown in Supplementary Figure 2, tacrolimus treatment at a dose of 10 mg/kg, which is higher than the doses (1–3 mg/kg, p.o., 10 days) used in the study for the femoral arterial ischemic reperfusion-induced NeuP model in rats (Muthuraman and Sood, 2010) at 30 min prior to the second acid injection, significantly inhibited mechanical allodynia at P5. Given that tacrolimus suppresses the activities of various immune cells, including macrophages and T cells (Yoshino et al., 2010; Kannegieter et al., 2018), these cells could be involved in the AcGP, yet the possibility cannot be excluded that tacrolimus exerts inhibitory actions via microglia in the central nervous system. The involvement of peripheral immune cells in AcGP was evidenced by the experiments that the injection of partially purified splenocytes from repeated acid-treated male and female mice into naive mice led to an induction of the abnormal mechanical allodynia and hypersensitive behaviors to 2,000 and 250 Hz electrical stimulation. The allodynia by splenocytes of male mice with AcGP gradually decreased day 5 and later, while the allodynia by female splenocytes was constant throughout day 7. It should be noted that the reproduction of allodynia by splenocytes of male mice with AcGP was abolished by 2nd-stage i.c.v. pretreatments with minocycline, while minocycline pretreatments did not affect the allodynia by female splenocytes. These findings suggest that repeated acid injections could drive the activation of peripheral immune system possibly via brain microglia in male mice, though it remains elusive how the activation of splenocytes in female mice is regulated. The direct activation of peripheral immune system by repeated acid injections or muscle insults cannot be excluded in both male and female mice. As there are many reports of the involvement of T cells in FM patients (Banfi et al., 2020), we attempted to further separate splenocytes into CD4^+^ T cells to see the reproduction of allodynia as a pioneering study, though we understand that the possible changes in the population of different T cell subsets should be analyzed as the next subject. Here, we observed the successful reproduction of allodynia by T cells.

The present study includes the pioneering finding that pain-related brain mechanisms are in part represented by peripheral immune mechanisms, though many intriguing questions remain unanswered. One important question is a type of peripheral immune cell from splenocytes involved in the generation of abnormal pain, and possible candidates are T lymphocytes and macrophage; both have sex-specific roles in NeuP (Sorge et al., 2015; Luo et al., 2019). Also, we need to identify the immune cell-derived factors that mediate AcGP. In this context, a recent paper has clarified neuronal circuits from corticotropin-releasing hormone-positive neurons within central amygdala and paraventricular hypothalamus to spleen, by using a combination of retrograde pseudo-rabies virus and optogenetic approach (Zhang et al., 2020). Further investigation of these animal models is essential to elucidate the mechanisms of sexual dimorphism in FM.

In conclusion, the present study demonstrates that repeated AcGP has a nature of generalized pain disease, accompanied by a type of sexual dimorphism, specifically after gonadectomy. We found another type of sexual dimorphism in AcGP in terms of microglia involvement. The repeated brain treatments with minocycline abolished the AcGP only in male mice and the reproduction of allodynia in naive mice by splenocytes derived from male but not female mice with AcGP. Purified CD4^+^ T cells from splenocytes were found to retain the ability to cause allodynia in naïve mice. These findings suggest that FM-like AcGP has multiple sexual dimorphic mechanisms.

## Data Availability Statement

The original contributions presented in the study are included in the article/Supplementary Material, further inquiries can be directed to the corresponding author.

## Ethics Statement

The animal study was reviewed and approved by Nagasaki University and Kyoto University Animal Research Committee.

## Author Contributions

ND, KT, and HN conducted experiments. ND, HN, and HUc performed data analysis. HUe and SK participated in research design. HUe and HUc wrote the manuscript. All authors contributed to the article and approved the submitted version.

## Conflict of Interest

The authors declare that the research was conducted in the absence of any commercial or financial relationships that could be construed as a potential conflict of interest.

## References

[B1] Albrecht D. S. Forsberg A. Sandström A. Bergan C. Kadetoff D. Protsenko E. (2019). Brain glial activation in fibromyalgia - A multi-site positron emission tomography investigation. *Brain Behav. Immun.* 75 72–83. 10.1016/j.bbi.2018.09.018 30223011 PMC6541932

[B2] Banfi G. Diani M. Pigatto P. D. Reali E. (2020). T Cell Subpopulations in the Physiopathology of Fibromyalgia: Evidence and Perspectives. *Int. J. Mol. Sci.* 21:1186. 10.3390/ijms21041186 32054062 PMC7072736

[B3] Boerner K. E. Chambers C. T. Gahagan J. Keogh E. Fillingim R. B. Mogil J. S. (2018). Conceptual complexity of gender and its relevance to pain. *Pain* 159 2137–2141. 10.1097/j.pain.0000000000001275 29781962

[B4] Chen G. Zhang Y. Q. Qadri Y. J. Serhan C. N. Ji R. R. (2018). Microglia in Pain: Detrimental and Protective Roles in Pathogenesis and Resolution of Pain. *Neuron* 100 1292–1311. 10.1016/j.neuron.2018.11.009 30571942 PMC6312407

[B5] Clauw D. J. (2014). Fibromyalgia: a clinical review. *JAMA* 311 1547–1555. 10.1001/jama.2014.3266 24737367

[B6] Costigan M. Moss A. Latremoliere A. Johnston C. Verma-Gandhu M. Herbert T. A. (2009a). T-cell infiltration and signaling in the adult dorsal spinal cord is a major contributor to neuropathic pain-like hypersensitivity. *J. Neurosci.* 29 14415–14422. 10.1523/jneurosci.4569-09.2009 19923276 PMC2813708

[B7] Costigan M. Scholz J. Woolf C. J. (2009b). Neuropathic pain: a maladaptive response of the nervous system to damage. *Annu. Rev. Neurosci.* 32 1–32. 10.1146/annurev.neuro.051508.135531 19400724 PMC2768555

[B8] Haley T. J. McCormick W. G. (1957). Pharmacological effects produced by intracerebral injection of drugs in the conscious mouse. *Br. J. Pharmacol. Chemother.* 12 12–15. 10.1111/j.1476-5381.1957.tb01354.x 13413144 PMC1509635

[B9] Häuser W. Ablin J. Fitzcharles M. A. Littlejohn G. Luciano J. V. Usui C. (2015). Fibromyalgia. *Nat. Rev. Dis. Primers* 1:15022. 10.1038/nrdp.2015.22 27189527

[B10] Häuser W. Ablin J. Perrot S. Fitzcharles M. A. (2017). Management of fibromyalgia: practical guides from recent evidence-based guidelines. *Pol. Arch. Intern. Med.* 127 47–56. 10.20452/pamw.3877 28075425

[B11] Inoue M. Yamaguchi A. Kawakami M. Chun J. Ueda H. (2006). Loss of spinal substance P pain transmission under the condition of LPA1 receptor-mediated neuropathic pain. *Mol. Pain* 2:25. 10.1186/1744-8069-2-25 16914035 PMC1562366

[B12] Ji R. R. Chamessian A. Zhang Y. Q. (2016). Pain regulation by non-neuronal cells and inflammation. *Science* 354 572–577. 10.1126/science.aaf8924 27811267 PMC5488328

[B13] Ji R. R. Donnelly C. R. Nedergaard M. (2019). Astrocytes in chronic pain and itch. *Nat. Rev. Neurosci.* 20 667–685. 10.1038/s41583-019-0218-1 31537912 PMC6874831

[B14] Kannegieter N. M. Hesselink D. A. Dieterich M. de Graav G. N. Kraaijeveld R. Baan C. C. (2018). Analysis of NFATc1 amplification in T cells for pharmacodynamic monitoring of tacrolimus in kidney transplant recipients. *PLoS One* 13:e0201113. 10.1371/journal.pone.0201113 30036394 PMC6056039

[B15] Khasar S. G. Dina O. A. Green P. G. Levine J. D. (2009). Sound stress-induced long-term enhancement of mechanical hyperalgesia in rats is maintained by sympathoadrenal catecholamines. *J. Pain* 10 1073–1077. 10.1016/j.jpain.2009.04.005 19576859 PMC2757466

[B16] Khasar S. G. Miao F. J. Jänig W. Levine J. D. (1998). Vagotomy-induced enhancement of mechanical hyperalgesia in the rat is sympathoadrenal-mediated. *J. Neurosci.* 18 3043–3049. 10.1523/jneurosci.18-08-03043.1998 9526021 PMC6792577

[B17] Koga K. Furue H. Rashid M. H. Takaki A. Katafuchi T. Yoshimura M. (2005). Selective activation of primary afferent fibers evaluated by sine-wave electrical stimulation. *Mol. Pain* 1:13. 10.1186/1744-8069-1-13 15813963 PMC1083421

[B18] Kuner R. Flor H. (2016). Structural plasticity and reorganisation in chronic pain. *Nat. Rev. Neurosci.* 18 20–30. 10.1038/nrn.2016.162 27974843

[B19] Lesnak J. B. Inoue S. Lima L. Rasmussen L. Sluka K. A. (2020). Testosterone protects against the development of widespread muscle pain in mice. *Paindoi* 161 2898–2908. 10.1097/j.pain.0000000000001985 32658149 PMC7669728

[B20] Luo X. Huh Y. Bang S. He Q. Zhang L. Matsuda M. (2019). Macrophage Toll-like Receptor 9 Contributes to Chemotherapy-Induced Neuropathic Pain in Male Mice. *J. Neurosci.* 39 6848–6864. 10.1523/jneurosci.3257-18.2019 31270160 PMC6733562

[B21] Mapplebeck J. C. Beggs S. Salter M. W. (2016). Sex differences in pain: a tale of two immune cells. *Pain* 157 S2–S6. 10.1097/j.pain.0000000000000389 26785152

[B22] Mapplebeck J. C. Beggs S. Salter M. W. (2017). Molecules in pain and sex: a developing story. *Mol. Brain* 10:9. 10.1186/s13041-017-0289-8 28270169 PMC5341415

[B23] Matsumoto M. Inoue M. Hald A. Yamaguchi A. Ueda H. (2006). Characterization of three different sensory fibers by use of neonatal capsaicin treatment, spinal antagonism and a novel electrical stimulation-induced paw flexion test. *Mol. Pain* 2:16. 10.1186/1744-8069-2-16 16681855 PMC1482679

[B24] Matsumoto M. Xie W. Ma L. Ueda H. (2008). Pharmacological switch in Abeta-fiber stimulation-induced spinal transmission in mice with partial sciatic nerve injury. *Mol. Pain* 4:25. 10.1186/1744-8069-4-25 18620588 PMC2488330

[B25] Miyamoto K. Kume K. Ohsawa M. (2017). Role of microglia in mechanical allodynia in the anterior cingulate cortex. *J. Pharmacol. Sci.* 134 158–165. 10.1016/j.jphs.2017.05.010 28669596

[B26] Mogil J. S. (2020). Qualitative sex differences in pain processing: emerging evidence of a biased literature. *Nat. Rev. Neurosci.* 21 353–365. 10.1038/s41583-020-0310-6 32440016

[B27] Muthuraman A. Sood S. (2010). Pharmacological evaluation of tacrolimus (FK-506) on ischemia reperfusion induced vasculatic neuropathic pain in rats. *J. Brachial. Plex Peripher. Nerve. Inj.* 5:13. 10.1186/1749-7221-5-13 20529260 PMC2890670

[B28] Nagakura Y. Oe T. Aoki T. Matsuoka N. (2009). Biogenic amine depletion causes chronic muscular pain and tactile allodynia accompanied by depression: A putative animal model of fibromyalgia. *Pain* 146 26–33. 10.1016/j.pain.2009.05.024 19646816

[B29] Neyama H. Dozono N. Ueda H. (2020). NR2A-NMDA Receptor Blockade Reverses the Lack of Morphine Analgesia Without Affecting Chronic Pain Status in a Fibromyalgia-Like Mouse Model. *J. Pharmacol. Exp. Ther.* 373 103–112. 10.1124/jpet.119.262642 31941720

[B30] Nishiyori M. Ueda H. (2008). Prolonged gabapentin analgesia in an experimental mouse model of fibromyalgia. *Mol. Pain* 4:52. 10.1186/1744-8069-4-52 18990235 PMC2596100

[B31] Salter M. W. Stevens B. (2017). Microglia emerge as central players in brain disease. *Nat. Med.* 23 1018–1027. 10.1038/nm.4397 28886007

[B32] Scholz J. Woolf C. J. (2007). The neuropathic pain triad: neurons, immune cells and glia. *Nat. Neurosci.* 10 1361–1368. 10.1038/nn1992 17965656

[B33] Sluka K. A. Kalra A. Moore S. A. (2001). Unilateral intramuscular injections of acidic saline produce a bilateral, long-lasting hyperalgesia. *Muscle Nerve* 24 37–46. 10.1002/1097-4598(200101)24 11150964

[B34] Sophocleous A. Idris A. I. (2019). Ovariectomy/Orchiectomy in Rodents. *Methods Mol. Biol.* 1914 261–267. 10.1007/978-1-4939-8997-3_13 30729469

[B35] Sorge R. E. LaCroix-Fralish M. L. Tuttle A. H. Sotocinal S. G. Austin J. S. Ritchie J. (2011). Spinal cord Toll-like receptor 4 mediates inflammatory and neuropathic hypersensitivity in male but not female mice. *J. Neurosci.* 31 15450–15454. 10.1523/jneurosci.3859-11.2011 22031891 PMC3218430

[B36] Sorge R. E. Mapplebeck J. C. Rosen S. Beggs S. Taves S. Alexander J. K. (2015). Different immune cells mediate mechanical pain hypersensitivity in male and female mice. *Nat. Neurosci.* 18 1081–1083. 10.1038/nn.4053 26120961 PMC4772157

[B37] Ueda H. (2008). Peripheral mechanisms of neuropathic pain - involvement of lysophosphatidic acid receptor-mediated demyelination. *Mol. Pain* 4:11. 10.1186/1744-8069-4-11 18377664 PMC2365930

[B38] Ueda H. (2017). Lysophosphatidic acid signaling is the definitive mechanism underlying neuropathic pain. *Pain* 158 S55–S65. 10.1097/j.pain.0000000000000813 28151833

[B39] Ueda H. Neyama H. (2017). LPA1 receptor involvement in fibromyalgia-like pain induced by intermittent psychological stress, empathy. *Neurobiol. Pain* 1 16–25. 10.1016/j.ynpai.2017.04.002 31194005 PMC6550118

[B40] van Hecke O. Austin S. K. Khan R. A. Smith B. H. Torrance N. (2014). Neuropathic pain in the general population: a systematic review of epidemiological studies. *Pain* 155 654–662. 10.1016/j.pain.2013.11.013 24291734

[B41] Wolfe F. Clauw D. J. Fitzcharles M. A. Goldenberg D. L. Häuser W. Katz R. L. (2016). 2016 Revisions to the 2010/2011 fibromyalgia diagnostic criteria. *Semin. Arthritis. Rheum.* 46 319–329. 10.1016/j.semarthrit.2016.08.012 27916278

[B42] Wolfe F. Clauw D. J. Fitzcharles M. A. Goldenberg D. L. Häuser W. Katz R. S. (2011). Fibromyalgia criteria and severity scales for clinical and epidemiological studies: a modification of the ACR Preliminary Diagnostic Criteria for Fibromyalgia. *J. Rheumatol.* 38 1113–1122. 10.3899/jrheum.100594 21285161

[B43] Wolfe F. Clauw D. J. Fitzcharles M. A. Goldenberg D. L. Katz R. S. Mease P. (2010). The American College of Rheumatology preliminary diagnostic criteria for fibromyalgia and measurement of symptom severity. *Arthritis Care Res.* 62 600–610. 10.1002/acr.20140 20461783

[B44] Wolfe F. Häuser W. Walitt B. T. Katz R. S. Rasker J. J. Russell A. S. (2014). Fibromyalgia and physical trauma: the concepts we invent. *J. Rheumatol.* 41 1737–1745. 10.3899/jrheum.140268 25086080

[B45] Wolfe F. Ross K. Anderson J. Russell I. J. Hebert L. (1995). The prevalence and characteristics of fibromyalgia in the general population. *Arthritis. Rheum.* 38 19–28. 10.1002/art.1780380104 7818567

[B46] Wolfe F. Smythe H. A. Yunus M. B. Bennett R. M. Bombardier C. Goldenberg D. L. (1990). The American College of Rheumatology 1990 Criteria for the Classification of Fibromyalgia. *Rep. Mult. Crit. Commit. Arthr. Rheum.* 33 160–172. 10.1002/art.1780330203 2306288

[B47] Yardeni T. Eckhaus M. Morris H. D. Huizing M. Hoogstraten-Miller S. (2011). Retro-orbital injections in mice. *Lab. Anim.* 40 155–160. 10.1038/laban0511-155 21508954 PMC3158461

[B48] Yoshino T. Nakase H. Honzawa Y. Matsumura K. Yamamoto S. Takeda Y. (2010). Immunosuppressive effects of tacrolimus on macrophages ameliorate experimental colitis. *Inflamm. Bowel. Dis.* 16 2022–2033. 10.1002/ibd.21318 20848491

[B49] Yu X. Liu H. Hamel K. A. Morvan M. G. Yu S. Leff J. (2020). Dorsal root ganglion macrophages contribute to both the initiation and persistence of neuropathic pain. *Nat. Commun.* 11:264. 10.1038/s41467-019-13839-2 31937758 PMC6959328

[B50] Zhang X. Lei B. Yuan Y. Zhang L. Hu L. Jin S. (2020). Brain control of humoral immune responses amenable to behavioural modulation. *Nature* 581 204–208. 10.1038/s41586-020-2235-7 32405000

