# Supplementary material for: Allodynia by Splenocytes From Mice With Acid-Induced Fibromyalgia-Like Generalized Pain and Its Sexual Dimorphic Regulation by Brain Microglia
Source: Front Neurosci. 2020 Dec 23;14:600166. doi: 10.3389/fnins.2020.600166 (PMC7785978; doi:10.3389/fnins.2020.600166)
Supplement: Supplementary file 1 [file Data_Sheet_1.docx]

**
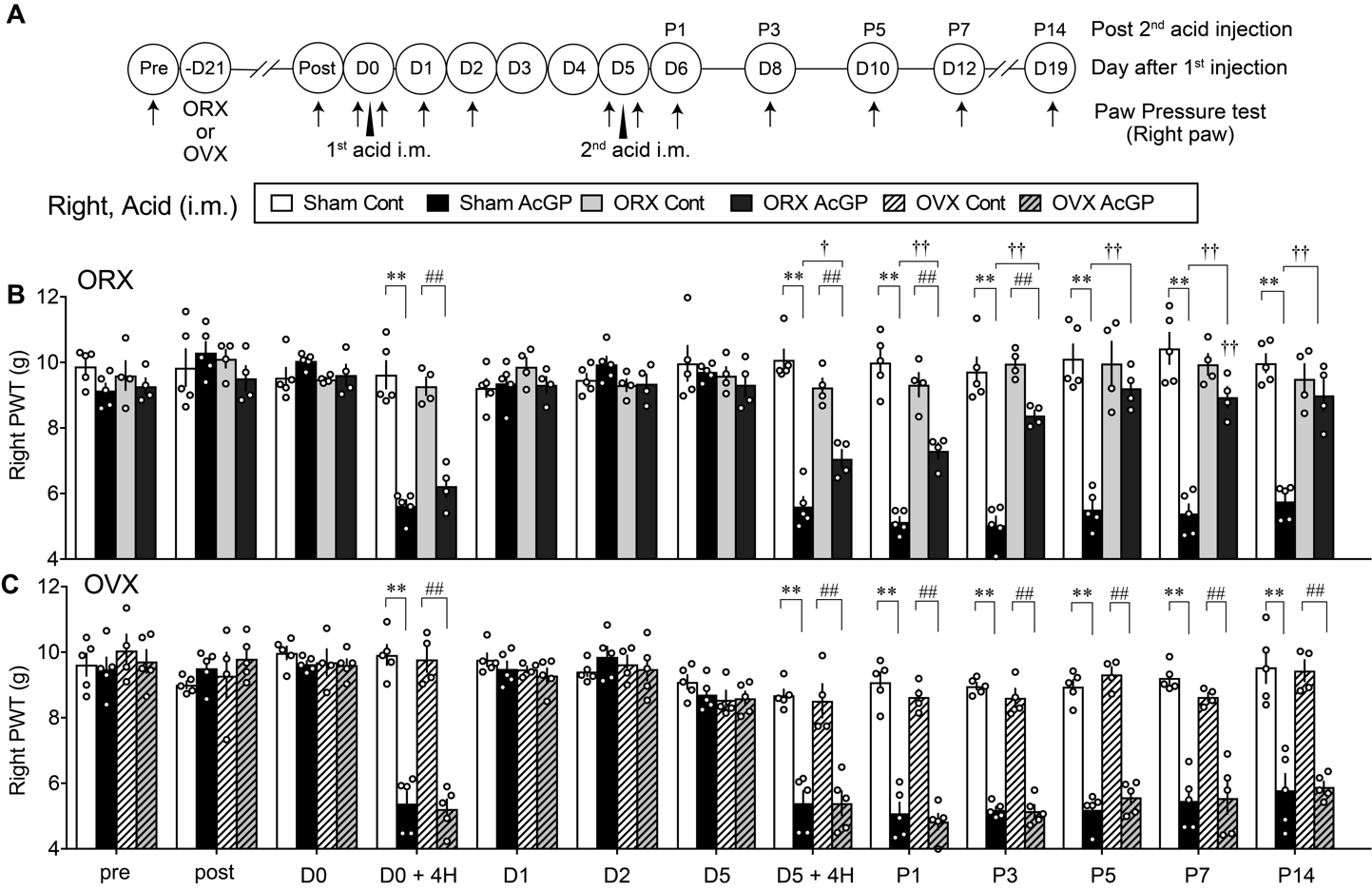
**

**FIGURE S1.** Male-specific blockade of ipsilateral AcGP by gonadectomy. **(A-C)** Details are described in the legend of Figure 4, except for the paw pressure test on the right side. ***p* < 0.01, compared with each time point at Sham Cont, ^#^*p* < 0.05; ^##^*p* < 0.01, compared with each time point at ORX Cont (B) or OVX Cont (C), ^†^*p* < 0.05; ^††^*p* < 0.01, compared with each time point at Sham AcGP, in two-way repeated measures ANOVA followed by Tukey’s multiple comparisons test (Sham Cont, n=4-5; Sham AcGP n=5; ORX Cont n=4, ORX Acid n=4; OVX Cont n=4; OVX AcGP n=5). The dots in the column represent number of animals. AcGP, Acid saline-induced fibromyalgia-like generalized pain; ORX, orchiectomy, OVX, ovariectomy; PWT, paw withdrawal threshold.


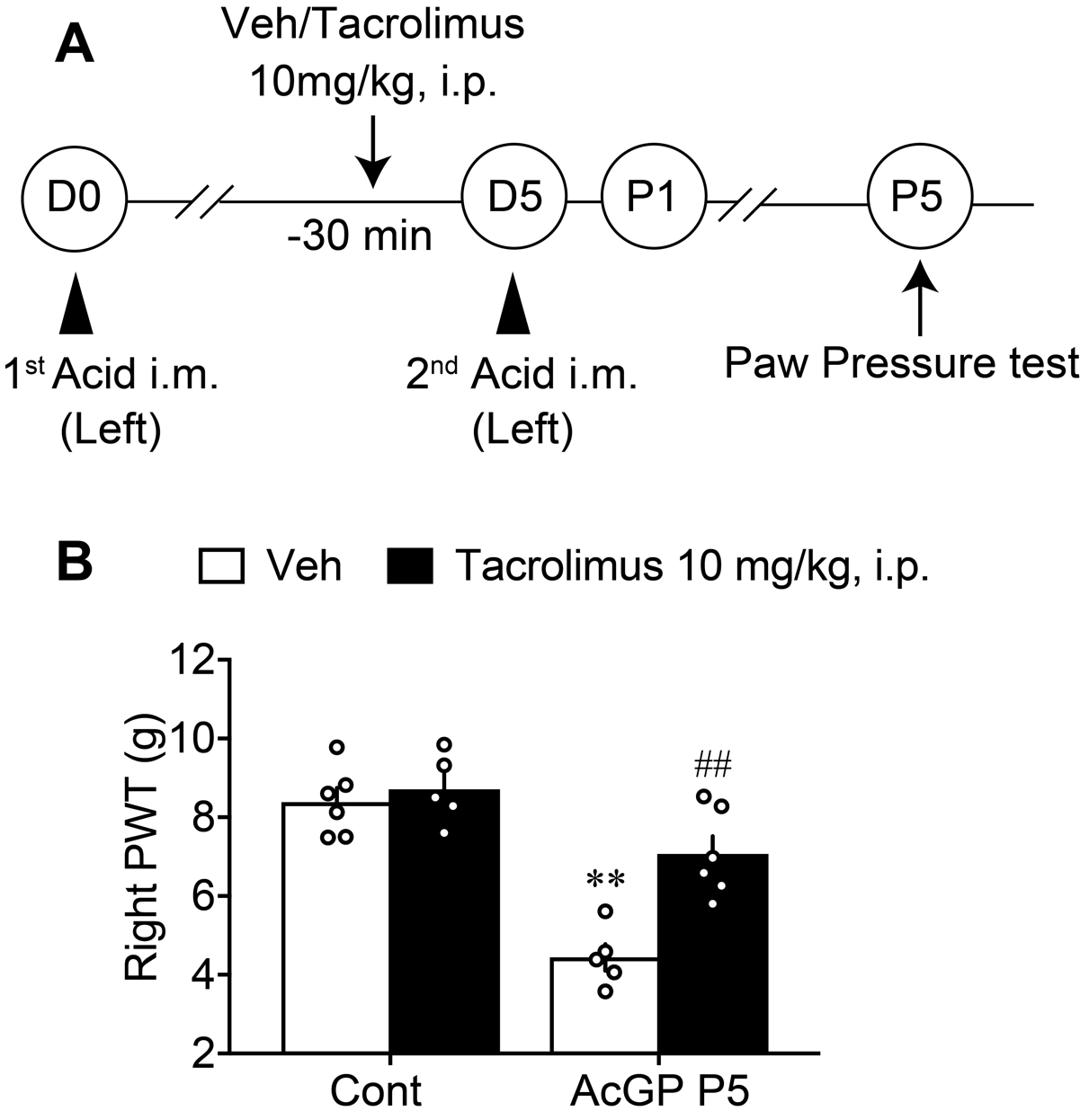


**FIGURE S2.** Inhibition of AcGP by tacrolimus. **(A)** Schedule of experimental design. Vehicle (saline) or tacrolimus at 10 mg/kg was administered 30 min before the second acid saline injection to the left muscle. Paw pressure test on the right side was performed at P5. **(B)** Inhibition of mechanical hyperalgesia in mice with AcGP by pretreatment with tacrolimus. ***p* < 0.01, compared with Cont Veh, ^##^*p* < 0.01, compared with AcGP Veh, in two-way ANOVA followed by Bonferroni’s multiple comparisons test (n=6). The dots in the column represent number of animals. AcGP, Acid saline-induced fibromyalgia-like generalized pain; PWT, paw withdrawal threshold.


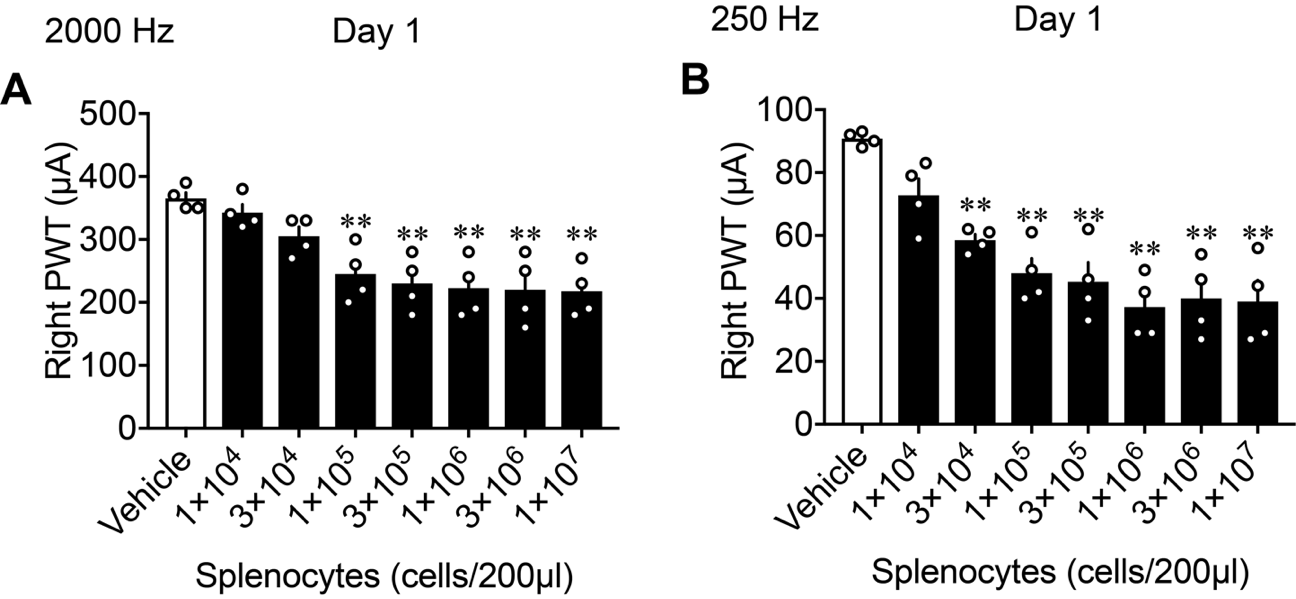


**FIGURE S3.** Cell number-dependent hyperalgesia by splenocytes from mice with AcGP. **(A,B)** Mechanical hyperalgesia by splenocytes in the EPW test. Results (PWT, μA) represent the thresholds of electric currents to cause pain-related paw withdrawal behaviors on the right side at day 1 after the i.v. injection of various number of splenocytes from male mice with AcGP into naïve male mice. Electrical stimulation at 2000 (**A**) and 250 Hz (**B**) was given to the right paw. Four independent experiments were performed. ***p* < 0.01, compared with Vehicle (PBS). PWT, paw withdrawal threshold.


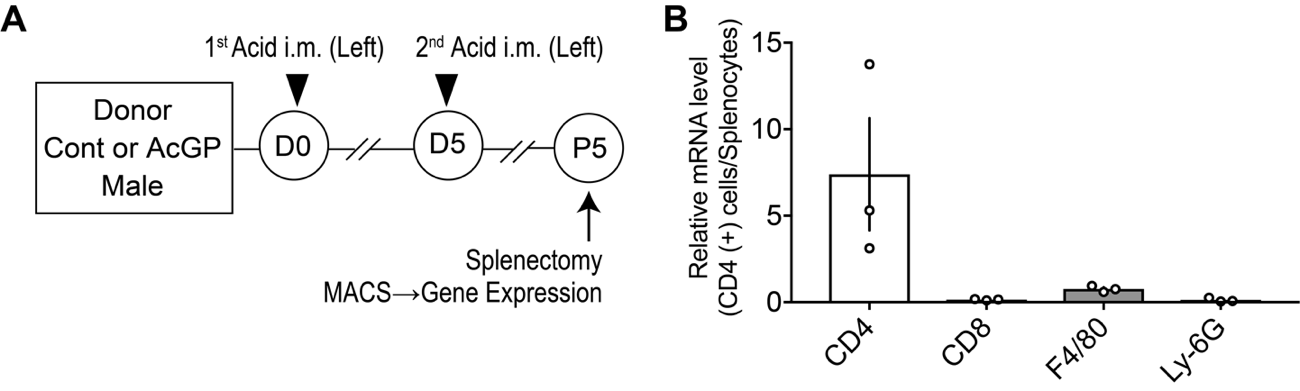


**FIGURE S4.** Purity of separated splenic CD4(+) T-cells. To check the purity, cDNA was synthesized from total RNA isolated from splenocytes and CD4(+) cells by using ReverTra Ace^®^ qPCR RT Master mix (TOYOBO, Osaka, Japan), and then used for quantitative real-time PCR. Real-time PCR was performed with THUNDERBIRD^®^ SYBR qPCR Mix (TOYOBO) and StepOnePlus Real-Time PCR System (Life Technologies, Carlsbad, CA, United States). Glyceraldehyde-3-phosphate dehydrogenase (GAPDH) was used as an internal control for normalization. The primer sequences were as follows: GAPDH, 5’-TGTCCGTCGTGGATCTGAC-3’ (forward), 5’-CCTGCTTCACCACCTTCTTG-3’ (reverse); CD4 (a T cell marker), 5’-ACACACCTGTGCAAGAAGCA-3’ (forward), 5’-GCTCTTGTTGGTTGGGAATC-3’ (reverse); CD8 (a cytotoxic T lymphocyte marker), 5’-GGCTCTGGCTGGTCTTCA-3’ (forward), 5’-GACGAAGGGGTCTGAATGAG-3’ (reverse); F4/80 (a macrophage marker), 5’-CCTGGACGAATCCTGTGAAG-3’ (forward), 5’- ATGAAGGTGGGACCACAGAG-3’ (reverse); Ly-6G (a neutrophil marker), 5’-TTGTGGTCCTACTGTGTGCAG-3’(forward), 5’-TCAGGTGGGACCCCAATAC-3’(reverse). In all cases, the validity of amplification was confirmed by the presence of a single peak in the melting temperature analysis and linear amplification with an increasing number of PCR cycles. Results represent the ratios of the transcription of CD4, CD8, F4/80 and Ly-6G to that of GAPDH in splenocytes and separated CD4^+^ cells.
